# Sample size and power considerations in network meta-analysis

**DOI:** 10.1186/2046-4053-1-41

**Published:** 2012-09-19

**Authors:** Kristian Thorlund, Edward J Mills

**Affiliations:** 1 Department of Clinical Epidemiology and Biostatistics, Faculty of Health Sciences, McMaster University, 1280 Main Street West, Hamilton, Ontario, Canada L8S 4 K1

**Keywords:** Network meta-analysis, Indirect comparison, Sample size, Power, Strength of evidence

## Abstract

**Background:**

Network meta-analysis is becoming increasingly popular for establishing comparative effectiveness among multiple interventions for the same disease. Network meta-analysis inherits all methodological challenges of standard pairwise meta-analysis, but with increased complexity due to the multitude of intervention comparisons. One issue that is now widely recognized in pairwise meta-analysis is the issue of sample size and statistical power. This issue, however, has so far only received little attention in network meta-analysis. To date, no approaches have been proposed for evaluating the adequacy of the sample size, and thus power, in a treatment network.

**Findings:**

In this article, we develop easy-to-use flexible methods for estimating the ‘effective sample size’ in indirect comparison meta-analysis and network meta-analysis. The effective sample size for a particular treatment comparison can be interpreted as the number of patients in a pairwise meta-analysis that would provide the same degree and strength of evidence as that which is provided in the indirect comparison or network meta-analysis. We further develop methods for retrospectively estimating the statistical power for each comparison in a network meta-analysis. We illustrate the performance of the proposed methods for estimating effective sample size and statistical power using data from a network meta-analysis on interventions for smoking cessation including over 100 trials.

**Conclusion:**

The proposed methods are easy to use and will be of high value to regulatory agencies and decision makers who must assess the strength of the evidence supporting comparative effectiveness estimates.

## Background

Over the past 2 decades, meta-analysis has become increasingly accepted by clinicians, decision-makers and the public as providing high-quality assessments of evidence [1]. Network meta-analysis, a new expansion of meta-analysis that allows for simultaneous comparison of several treatments, is similarly becoming increasingly accepted in the clinical research community [2-11]. Having been available for more than 3 decades, meta-analysis has been studied extensively, and several hundred articles published in this period have identified and resolved a vast array of basic and advanced methodological issues [1,12]. Network meta-analysis inherits all the challenges present in a standard meta-analysis (e.g., issues of bias, heterogeneity and precision), but with increased complexity due to the multitude of comparisons involved [5,13]. Since network meta-analysis is still a relatively new technique, the number of publications addressing methodological challenges is still relatively sparse.

One important issue that has received much attention in individual trials and meta-analysis is the issue of sample size and statistical power [14-26]. Several studies have demonstrated the importance of interpreting pooled meta-analysis estimates and confidence intervals according to the statistical level of evidence (i.e., precision) [14,15,18-24,26,27], and sound recommendations have been provided [16,22,28]. So far, however, only a small number of studies have addressed the issue of power and precision in network meta-analysis [8,13,29], and no comprehensive guidance exists on the topic. Network meta-analyses typically include many more trials than standard meta-analyses because of the multitude of comparisons involved and for this reason may artificially appear to provide a stronger evidence base. Likewise, the accompanying graphical representation of a treatment network can provide a similar compelling but potentially false impression of a strong evidence base.

Network meta-analysis utilizes evidence from direct (head-to-head) comparisons (i.e., trials directly comparing treatment A and B) and indirect comparisons (e.g., the combination of trials comparing A with C and trials comparing B with C) [4,6,30]. The major challenge in interpreting the power and precision of a network meta-analysis stems from the fact that there are (typically) varying levels of power and precision across all comparisons. In addition, the power and precision of indirect evidence are more complex to assess than for direct evidence, and thus, without proper guidance, it will be difficult for most authors to evaluate the precision gain from use of indirect evidence as well as the strength of evidence in a treatment network.

In this article, we provide guidance on quantifying the power and precision in network meta-analysis using simple sample size considerations. We first describe how to quantify the precision in indirect comparison meta-analysis and subsequently in network meta-analysis with combinations of direct and indirect evidence. We then outline the concept of sample size requirements and power calculations in pairwise meta-analysis. Finally, we show how to combine these measures in order to quantify the power and strength of evidence available for all treatment comparisons in a network meta-analysis. We illustrate the described methods using data from a recent network meta-analysis on interventions for smoking cessation [31].

## Methods

### Basic methodological framework

#### Indirect comparisons

Indirect effect estimates are obtained with the effect estimates from two comparisons sharing a common comparator [32]. For example, when two treatments A and B have both been compared to some common comparator C (e.g., placebo) in a number of randomized clinical trials, an indirect effect estimate of treatment A versus B can be obtained using the meta-analysis effect estimate of A versus C and the meta-analysis effect estimate of B versus C [32]. In particular, the indirect effect estimate of A versus B (*d*_*AB*_) is calculated as the estimated effect of A versus C (*d*_*AC*_) minus the estimated effect of B versus C (*d*_*BC*_). Mathematically, this corresponds to the equation

$$d_{\mathrm{AB}}=d_{\mathrm{AC}}-d_{\mathrm{BC}}\text{.}$$

(Note, when dealing with ratio effect measures, such as relative risks and odds ratios, all calculations are done on the log scale to preserve linearity and approximate normality). To produce confidence intervals for the indirect estimate, we first need to estimate its variance. The variance of in the indirect estimate of A versus B (*V*_*AB*_) is simply equal to the sum of the variance of the effect estimate of A versus C (*V*_*AC*_) and the variance of the effect estimate of A versus B (*V*_*AB*_). Mathematically this corresponds to the equation *V*_*AB*_ = *V*_*AC*_ + *V*_*BC*_. It is therefore clear that the variance of a (direct) meta-analysis effect estimate based on some number of trials, say *k*, will always be smaller than the variance of an indirect meta-analysis based on the same number of trials, *k* (all trial sample sizes being equal). In other words, direct estimates come with higher precision and power (trial count and trial sample sizes being equal). In many situations, however, using indirect estimates can add considerable power and precision.

### ***Combining direct and indirect evidence***

When both direct and indirect evidence is available, it may often be advantageous to combine the two statistically [2,4-7,30,33]. For example, if only two small trials have investigated two active interventions A and B head to head, but 20 trials have compared A or B with placebo, the indirect evidence will be able to add much power and precision to the comparative estimate of A and B. The combination of indirect and direct evidence requires advanced statistical regression techniques (i.e., network meta-analysis) that are beyond the scope of this article [4,6,30]. However, in the context of sample size and power considerations, it suffices to understand that indirect evidence, when combined with direct evidence, increases the power and precision of treatment effect estimates [4,6,7,9,30,33]. The extent to which is does so can be evaluated readily by using the methods we describe below.

### ***Sample size in indirect comparisons***

In this section we introduce three methods for gauging how much statistical precision an indirect estimate provides when no direct evidence is available. In particular, we describe how to approximate the amount of information required in a direct (head-to-head) meta-analysis to produce the same precision as that in the available indirect evidence. Simply put, what direct meta-analysis sample size would provide a similar degree of information? We dub this the *effective sample size* of the indirect evidence or, interchangeably, the *effective indirect sample size*. We describe three different methods for approximating the effective indirect sample size. Each of these methods differs with respect to simplicity and validity (the simpler one being the least valid), so we outline the simplicity-validity trade-offs at the end of the section.

### Method 1: the effective number of trials

A simple approach to gauging the degree of power and precision available in indirect evidence is to approximate how many trials are required in an indirect comparison to produce a matching degree of power and precision from a single head-to-head trial. This type of approximation is possible under the simple assumptions that the variances (of the mean) are equal for each trial and that no heterogeneity is present. Glenny et al. showed that when the number of trials is the same in both of two comparisons informing the indirect evidence (e.g., two trials of A vs. C and two trials of B vs. C), it takes four trials in the indirect evidence to produce the same precision as one direct head-to-head trial [8]. In indirect comparisons, however, it is common that one comparison will include more trials than the other. When this happens, the above 1:4 *precision ratio* no longer holds true. For example, if the number of trials is twice as high in one comparison (i.e., a 1:2 *trial count ratio*), the indirect comparison will need exactly 4.5 trials to produce the same precision as one head-to-head trial (see mathematical derivation in Appendix 1.a). In reality, however, to maintain a ratio of 1:2 in the trial count, one would need six trials (2:4) in the indirect comparison to produce the same precision as one head-to-head trial. To produce the same precision as two head-to-head trials, one would need 2 × 4.5 = 9 trials, which allows maintaining the 1:2 ratio with three trials in one comparison and six in the other (i.e., 3:6 as the trial count ratio). Table 1 presents the approximate number of trials required in an indirect comparison under different scenarios where the number of trials in the two comparisons is unbalanced. The mathematical derivations for all exact precision ratios are presented in Appendix 1.a.

**Table 1:** The required number of indirect comparison trials required to produce the same precision as a given number of direct (head-to-head) trials

**Trial count ratio**	**Exact precision ratio**	**Number of indirect comparison trials required to match precision from the corresponding number of single trials in a pair wise meta-analysis**					
		**1**	**2**	**3**	**4**	**5**	**10**
1:1	4	*4 (2:2)*	*8 (4:4)*	*12 (6:6)*	*16 (8:8)*	*20 (10:10)*	*40 (20:20)*
1:2	4.5	6 (2:4)	*9 (3:6)*	15 (5:10)	*18 (6:12)*	24 (8:16)	*45 (15:30)*
1:3	5.33	8 (2:6)	12 (3:9)	*16 (4:12****)***	24 (6:18)	28 (7:21)	56 (14:42)
1:4	6.25	10 (2:8)	15 (3:12)	20 (4:16)	*25 (5:20)*	35 (7:28)	65 (13:52)
1:5	7.2	12 (2:10)	18 (3:15)	24 (4:20)	30 (5:25)	*36 (6:30)*	*72 (12:60)*
1:6	8.17	14 (2:12)	21 (3:18)	28 (4:24)	35 (5:30)	42 (6:36)	84 (12:72)
1:7	9.14	16 (2:14)	24 (3:21)	32 (4:28)	40 (5:35)	48 (6:42)	96 (12:84)
1:8	10.13	18 (2:16)	27 (3:24)	36 (4:32)	45 (5:40)	54 (6:48)	108 (12:96)
1:9	11.11	20 (2:18)	30 (3:27)	40 (4:36)	50 (5:45)	60 (6:54)	120 (12:108)
1:10	12.1	22 (2:20)	33 (3:30)	44 (4:40)	55 (5:50)	66 (6:60)	*121 (11:110)*

The cells in underlined italics indicate where the indirect evidence produces the exact precision of the corresponding number of trials. The remaining cells indicate where the indirect evidence produces precision slightly above that of the corresponding number of head-to-head trials.

In some cases the required number of trials in an indirect comparison for a specific trial count ratio produces a precision corresponding to more than that of a stated number of single head-to-head trials. For example, with a trial count ratio of 1:3, one would require 2 × 5.33 = 10.66 indirect comparison trials to produce the precision of two head-to-head trials. However, since we cannot have fractions of trials, we take the closest integer above 10.66 where the trial count ratio is maintained: 12 trials with a trial count ratio of 3:9.

Table 1 can readily be used for quickly and easily checking how many head-to-head trials the indirect evidence ‘effectively’ corresponds to. That is, if the indirect evidence produces the precision of, say, three trials, we can think of the evidence as being as strong as a meta-analysis of three head-to-head trials. For example, if one has an indirect comparison with 4 trials comparing A with C, and 12 trials comparing B with C, the precision of the indirect comparison corresponds to a meta-analysis of 3 trials directly comparing A with B (Table 1). It should be noted that Table 1 is only valid to the extent that trial sample sizes and trial population variances are similar across trials, as well as the extent to which heterogeneity is absent or ignorable.

### Method 2: the effective sample size

Another relatively simple approach to gauging the degree of power and precision from an indirect comparison is to consider the collection of trials included in each comparison as one (large) clinical trial. From a sample size perspective, following similar mathematic derivations as the above trial count perspective, the relationship between the precision of an indirect comparison and the precision of a direct meta-analysis turns out the same (see Appendix 1.b for mathematical derivations). For example, to produce the same precision as a head-to-head meta-analysis including 1,000 patients, one would need a total of 4,000 (4 × 1,000) patients in the indirect comparison, provided the number of patients is the same for the two comparisons (2,000:2,000). Taking the sample size perspective comes with the flexibility of a possible reversal of the calculation. For example, an indirect comparison with 500 patients in the A vs. C comparison and 500 patients in the B vs. C comparisons would produce the same precision as a direct comparison with 250 patients [(500 + 500)/4]. Likewise, in a scenario with 1,000 patients in comparison A vs. C, and 10,000 patients in comparison B vs. C, the exact precision ratio is 12.1 (see Table 1), and so the effective direct meta-analysis sample size would be (1,000 + 10,000)/12.1 = 909.

Often, the sample sizes in the two comparisons do not line up to produce the exact precision ratio presented in Table 1 and Table 2. Letting *n*_*AC*_ and *n*_*BC*_ denote the sample sizes for the comparisons of A vs. C and B vs. C, respectively, a more general formula for the effective indirect sample size is (see Appendix 1.b).

$$\left(n_{\mathrm{AC}}\times n_{\mathrm{BC}}\right)/\left(n_{\mathrm{AC}}+n_{\mathrm{BC}}\right)$$

**Table 2:** Effective heterogeneity-corrected sample sizes of indirect comparison scenarios with varying degrees of patient count ratios and heterogeneity in each comparison (A vs. C and B vs. C), but with fixed total sample size of 10,000

**Patient ratio**	**Precision ratio**	**I**_**AC**_^**2**^	**I**_**BC**_^**2**^	**I**^**2**^**adjusted precision ratio**	**Effective sample size**
5,000:5,000 (1:1)	4.00	0%	0%	4.00	2,500
		0%	25%	4.60	2,188
		0%	50%	5.33	1,875
		25%	50%	6.40	1,563
		50%	50%	8.00	1,250
3,333:6,667 (1:2)	4.50	0%	0%	4.50	2,222
		0%	25%	5.40	1,852
		0%	50%	6.75	1,481
		25%	0%	4.91	2,037
		25%	25%	6.00	1,667
		25%	50%	7.72	1,296
		50%	0%	5.40	1,852
		50%	25%	6.75	1,482
		50%	50%	9.00	1,111
2,500:7,500 (1:3)	5.33	0%	0%	5.33	1,876
		0%	25%	6.56	1,524
		0%	50%	8.53	1,173
		25%	0%	5.69	1,759
		25%	25%	7.11	1,407
		25%	50%	9.48	1,055
		50%	0%	6.09	1,642
		50%	25%	7.75	1,290
		50%	50%	10.7	938
2,000:8,000 (1:4)	6.25	0%	0%	6.25	1,600
		0%	25%	7.81	1,280
		0%	50%	10.4	960
		25%	0%	6.58	1,520
		25%	25%	8.33	1,200
		25%	50%	11.4	880
		50%	0%	6.94	1,440
		50%	25%	8.93	1,120
		50%	50%	12.5	800
1,667:8,333 (1:5)	7.20	0%	0%	7.20	1,389
		0%	25%	9.09	1,100
		0%	50%	12.4	810
		25%	0%	7.51	1,331
		25%	25%	9.60	1,042
		25%	50%	13.3	752
		50%	0%	7.86	1,273
		50%	25%	10.2	984
		50%	50%	14.4	694
1,000:9,000 (1:9)	11.11	0%	0%	11.1	900
		0%	25%	14.3	698
		0%	50%	20.2	495
		25%	0%	11.4	878
		25%	25%	14.8	675
		25%	50%	21.1	473
		50%	0%	11.7	855
		50%	25%	15.3	653
		50%	50%	22.2	450

For the above example, the effective sample size using this formula is therefore $\left(1000\times 10000\right)/\left(1000+10000\right)=909\text{.}$

The above simple approach to calculate the effective indirect sample size does not consider the possibility that statistical heterogeneity exists across trials. When heterogeneity is present, the effect estimates that go into the indirect estimate incur a higher degree of variation, and so the effective indirect sample size corresponding to a head-to-head meta-analysis will be smaller than with the above simple approach. In line with already-established heterogeneity corrections for meta-analysis required sample sizes [23,24,28], we put forward that the actual number of patients in each of the comparisons informing the indirect estimate can be penalized by the additional variation explained by heterogeneity. In line with previous proposals, we penalize for the ‘lack of homogeneity’ [23,24] using the popular measure of heterogeneity, *I*^*2*^[34], as a basis for the penalization.

Consider the example where a meta-analysis of A vs. C includes 6,000 patients and a meta-analysis of B vs. C includes 8,000 patients, and assume the estimated degree of heterogeneity for A vs. C is 50% (*I*_*AC*_^*2*^ *=* 50%) and 25% for B vs. C (*I*_*BC*_^*2*^ *=* 25%). Then the lack of homogeneity is 100%-50% = 50% for A vs. C and 100%-25% = 75% for B vs. C. We penalize the actual sample size by multiplying the actual sample size by the lack of homogeneity, so that the penalized sample size of A vs. C is 50% × 6,000 = 3,000, and the penalized sample size for B vs. C is 75% × 8,000 = 6,000. The total penalized number of patients in the indirect comparison is then 3,000 + 6,000 = 9,000, the patient count ratio is 1:2 (same as 3,000:6,000), the precision ratio is 4.5 (see Table 1), and so the effective heterogeneity-corrected sample size in this indirect comparison is 9,000/4.5 = 2,000.

Following the above example, the general formula for a heterogeneity-corrected effective sample size for indirect evidence is

$$\left(n_{\mathrm{AC}}\times \left(1-{I_{\mathrm{AC}}}^{2}\right)+n_{\mathrm{BC}}\times \left(1-{I_{\mathrm{BC}}}^{2}\right)\right)/\text{Precision ratio}$$

where *n*_*AC*_ and *n*_*BC*_ are the actual sample sizes (before correction) in the meta-analyses of A vs. C and B vs. C, respectively, and where the precision ratio is based on the heterogeneity-corrected sample sizes (see Table 1 and Table 2). In the appendix we provide a general formula for the precision ratio.

As with the above example of non-penalized sample sizes, the penalized sample sizes may not always line up to match the precision ratio given in Table 1 and Table 2. The more general formula for the heterogeneity-corrected effective sample size is (see Appendix 1.b).

$$\left[\left(n_{\mathrm{AC}}\times \left(1-{I_{\mathrm{AC}}}^{2}\right)\right)\times \left(n_{\mathrm{BC}}\times \left(1-{I_{\mathrm{BC}}}^{2}\right)\right)\right]/\left[n_{\mathrm{AC}}\times \left(1-{I_{\mathrm{AC}}}^{2}\right)\right)+\left(n_{\mathrm{BC}}\times \left(1-{I_{\mathrm{BC}}}^{2}\right)\right)]$$

One immediate limitation of the above-proposed sample size heterogeneity correction is the fact that *I*^*2*^s are typically unreliable and unstable in meta-analyses including a limited number of trials and will depend on the effect metric used [34-36]. In most cases, it will therefore be preferable to simply assume some plausible degree (percentage) of heterogeneity derived from a mix of clinical considerations and the I^2^ estimate at hand. Typically, an assumption of 25% or 50% heterogeneity will be reasonable in the context of sample size considerations [22]. Table 2 illustrates the effective sample size for various indirect comparison scenarios including a total of 10,000 patients, under different combinations of heterogeneity corrections (one including no correction).

Another limitation of the sample size approach is the inherent assumption that sample size is a good proxy for precision. This may not be true if there are some important differences in event rates (for binary data) or counts (count data), or if there are population differences in trials that result in notably different standard deviations, but not necessarily different effect estimates. To curb this limitation for binary data, one may for example choose to focus on the effective number of events. A more universally applicable approach focuses on a measure called *statistical information*. We describe this below.

### The effective statistical information

Statistical information, also sometimes referred to as Fisher information, is a more complex statistical measure for gauging the degree of precision present in a data set. For pairwise meta-analyses, the statistical information is equal to the inverse of the pooled variance (i.e., one divided by the variance), which is also the measure of precision [37]. For indirect comparisons, the statistical information (precision) is equal the inverse of the pooled indirect variance. That is, with variances *V*_*AC*_ and *V*_*BC*_ for comparisons A vs. C and B vs. C, the indirect variance is *V*_*AC*_ + *V*_*BC*_*,* so indirect statistical information is 1/(*V*_*AC*_ + *V*_*BC*_). Because the variance incorporates heterogeneity and is dependent on the number of trials and the sample size, no further calculations or adjustments are needed.

A major disadvantage of statistical information in meta-analysis is that it operates on a scale that no statistically non-sophisticated reader could easily grasp [38]. The statistical information may however be useful in the context of sample size requirements since it is possible to calculate the required statistical information (analogous to the required sample size) and compare the actual statistical information present in an indirect comparison with such a yardstick for sufficient power.

### Strength and limitations of the approaches

Each of the above approaches comes with strengths and limitations. These are outlined in Table 3.

**Table 3:** Strengths and limitations of the three approaches for gauging the effective degree of power and precision in indirect comparisons

**Approach**	**Strengths**	**Limitations**
Effective number of trials	1. Easy and fast to calculate	1. Only valid to the extent trial sample sizes are equal and heterogeneity is absent
		2. Lacks flexibility for approximate trial count ratios
Effective sample size (ignoring heterogeneity)	1. Easy and fast to calculate	1. Does not account for heterogeneity
	2. Exact calculations for all trial count ratios	2. Assumes equal meta-analysis population variances across comparisons
	3. Sample size (no. of patients) resonates well with clinicians	
Effective sample size (correcting for heterogeneity)	1. Exact calculations for all precision ratios	1. Assumes equal meta-analysis population variances across comparisons
	2. Accounts for heterogeneity	2. Depends on precise heterogeneity estimation
	3. Easy to calculate	
	4. Sample size (no. of patients) resonates well with clinicians	1. Statistical information does not resonate well with a clinical audience
Effective statistical information	1. Theoretically statistically exact	
		2. Not straight forward to calculate
		3. Depends on precise heterogeneity variance estimation or requires elicitation of Bayesian variance priors

### Weak links

So far we have only described situations where the indirect estimate is obtained through one common comparator. However, it is worth noting that indirect evidence may often come from scenarios where the link between two treatments of interest must go through two or more comparators. For example, if we wish to compare A with D and the following three comparisons are available—A vs. B, B vs. C and C vs. D—then the link between A and D goes through both B and C. The indirect variance is now a sum of three (rather than two) direct variances, $V_{\mathrm{AB}}+V_{\mathrm{BC}}+V_{\mathrm{CD}}$*.* We would therefore expect comparably smaller precision. Calculating the effective number of trials in the two common comparator example, we find that with an equal number of trials, say 3:3:3, the precision of the indirect comparison corresponds to that of only one direct trial, that is, an exact precision ratio of 1:9. With a more unbalanced number of trials, say 8:1:8, we get an exact precision ratio of 1:21.

Such consistently large direct to indirect precision ratios indicate that indirect comparisons with two (or more) comparators in the link will typically add very little precision. For this reason we dub them ‘weak links.’ In the context of combining direct and indirect evidence as we address below, it seems that weak links will typically only add an ignorable small amount of precision to the final estimate and thus, for simplicity, may be ignored for sample size and power considerations in indirect comparisons and network meta-analysis.

### Effective sample size in treatment networks

#### The three-treatment loop

The simplest example of a combination of direct and indirect evidence is the three-treatment loop where precision is added to the comparison of A and B by borrowing strength from an indirect comparison based on some common comparator C. Whether our measure of precision (information) is the number of trials, the sample size or the statistical information, the total amount of precision available for a particular comparison in a three-treatment loop is conceptually the sum of information in the direct evidence and in the indirect evidence.

To calculate the effective number of trials informing a particular comparison (e.g., A vs. B) in the combined evidence, we simply add the effective number of trials in the indirect evidence to the number of trials in the head-to-head evidence.

To calculate the effective number of patients informing a particular comparison, we simply add the effective number of patients in the indirect evidence to the number of patients in the head-to-head evidence. If heterogeneity adjustments have been applied for the indirect evidence, a similar adjustment should be applied to the direct evidence (we illustrate this in our worked example below).

To calculate the statistical information informing a particular comparison, we simply take the sum of the inverse indirect variance and the inverse direct variance. Alternatively, the statistical information may be extracted directly from the statistical software package used to run the network meta-analysis. (Note that if multi-arm trials are included in the analysis, some adjustments for correlations are needed).

#### Multiple sources of evidence

In many treatment networks two or more sources of indirect evidence informing some (or all) of the comparisons may exist. For example, two active treatments A and B may both have been compared to standard-of-care and placebo. In this case, indirect evidence exists from two sources. Extending the three-treatment example above, estimating the total effective amount of information is simply a task of summing all indirect and direct evidence.

In a similar situation, multiple sources of indirect evidence may exist where no direct evidence exists. In this case, the effective number of trials, sample size or statistical information is simply obtained by summing all indirect information.

We previously discussed ‘weak links.’ Since these add a relatively small amount of information, one may chose to ignore them without notable loss of information (but with ease in the calculations). This goes for both situations where direct evidence and indirect evidence are being combined, and where there are multiple sources of indirect evidence.

#### Power and sample size requirements in network meta-analysis

Before getting into sample size and power considerations for indirect comparison meta-analysis and network meta-analysis, we first outline the already well-established framework for pairwise meta-analysis. We then extend the concept to indirect comparison meta-analysis and lastly to network meta-analysis.

#### Sample size requirements for direct meta-analysis

Several methodological studies have explored sample size and power considerations for direct (head-to-head) meta-analysis [14,15,19-24,26]. By now, it has been well established that the required sample size (i.e., the required number of patients) for a meta-analysis, should be at least that of a large well-designed clinical trial [16]. Sample size calculations are derived from an a priori estimate of a treatment effect, *d*, that investigators wish to demonstrate; the associated variance around that treatment effect, *V*^*2*^; and a maximum risk of type I error, *α*, (i.e., maximum false-positive risk) and type II error, *β*, (i.e., maximum false-negative risk). As a basis, one can use the required sample size corresponding to a large multicenter clinical trial as the required sample size for the head-to-head meta-analysis [16,19,20,23,24].

$$N=C\times {\left(z_{1-}{{}_{a}}_{/2}+{z_{1-}}_{b}\right)}^{2}\times V^{2}/d^{2}$$

Here *z*_*1-α/2*_ and *z*_*1-β*_ are the (1-*α/2*)th and (1-*β*)th percentiles of a standard normal distribution, and *C* is a constant depending on the randomization ratio and number of treatment arms (C = 4 with a randomization ratio of 1:1 and two treatment arms).

If statistical heterogeneity exists across the included trials in a meta-analysis, one can adjust the calculated sample size to account for the additional variation (i.e., increased uncertainty) [21-24,28]. This is achieved by multiplying the required sample size, *N*, by a heterogeneity correction factor 1/(1-*H*), where *H* has a similar interpretation as the well-known measure *I*^*2*^ (the percentage of variation in a meta-analysis explained by heterogeneity) and is the a priori or maximum acceptable degree of heterogeneity [21-24,28]. Empirical evidence and simulations have demonstrated that such adjustments perform well in maintaining the desired statistical power [21,22,24].

An alternative approach to dealing with heterogeneity is to calculate the required *statistical information* (also known as Fisher information) [17,25]. In pairwise meta-analysis the statistical information is simply the inverse of the pooled variance, that is, the pooled precision. The required statistical information resembles the required sample size.

$$RI=C\times {\left(z_{1-}{{}_{a}}_{/2}+{z_{1-}}_{b}\right)}^{2}/d^{2}$$

A simulation study has demonstrated adequate performance of the required statistical information when the heterogeneity is modeled with certain Bayesian priors [17].

#### Information fractions and power in direct meta-analysis

At any point in a direct meta-analysis before the cumulative amount of evidence has surpassed the required sample size (or required statistical information), we can calculate two useful measures to gauge the strength of evidence. The first measure, the information fraction (IF), is the accrued number of patients, *n* (or statistical information), divided by the required sample size (or required statistical information) [19,23].

IF=n/N

This measure gives us an idea of how far we have come and how much farther of a distance there is to the yardstick—our required sample size.

The second measure is a retrospective power calculation. Re-arranging the expression of the required sample size, we can retrospectively estimate the power (*1-β*) of the current data

$$1-\beta =\Phi^{-1}\left(-z_{1-}{{}_{a}}_{/2}+\sqrt{\left(n\cdot d^{2}\right)\left(C\cdot V^{2}\right)}\right)$$

where *Φ* is the cumulative standard normal distribution function.

#### Information fractions and power in indirect comparisons

So far we have described method for estimating the effective sample size in indirect and combined evidence, as well as methods for estimating the required sample size and gauging the strength of evidence in pairwise meta-analyses. We now combine these measures to evaluate the strength of indirect comparisons and network meta-analyses. For the remainder of this article we concentrate on the number of patients, but our example and calculations can easily be carried out using the number of trials or statistical information.

We have introduced the effective sample size for indirect evidence. In line with its definition, we can derive the effective indirect information fraction and the effective power in an indirect comparison. The steps required to do this are as follows. First, calculate the effective indirect sample size. Second, calculate the required sample size for a direct meta-analysis. Third, to get the effective indirect information fraction, simply divide the effective number of patients in the indirect comparison by the required sample size for a direct comparison. Fourth, to calculate the power of the available indirect evidence, simply insert the effective indirect sample size as the *n* in the above formula.

#### Information fractions and power in network meta-analysis

The steps required to calculate the information fraction and power of treatment comparisons that are both informed by direct and indirect evidence or by multiple sources of indirect evidence are similar to the step required for indirect comparisons. First, calculate the effective sample size for the comparison of interest by summing up the evidence from the available sources (we described how to do this above). Second, as before, calculate the required sample size for a direct meta-analysis. Third, as before, calculate the effective information fraction by dividing the effective number of patients by the required sample size. Fourth, calculate the power of the available evidence by inserting the effective sample size as the *n* in the above formula.

## Results and discussion

### Worked example – interventions for smoking cessation

A recent MTC explored the efficacy of five different interventions for smoking cessation, low-dose NRT (<22 mg nicotine patches), which is an over-the-counter intervention, and four newer and more expensive interventions, combination NRT (i.e., patch plus gum), high-dose NRT (>22 mg patches), buproprion and varenicline [31].The published MTC provides a useful data-set to illustrate the issues we have raised in this article. Low-dose NRT is already well established and well known to yield about a 50% higher success rate than inert control interventions (i.e., relative risk of approximately 1.50). To consider any of the four newer treatments worthwhile, one could argue for the need to demonstrate at least an additional 20% efficacy. These considerations make up the basis for the required sample size considerations. Keep in mind that the published MTC has much higher rates of effective sample size than the assumptions we make in this example. [31]

**Figure 1:**
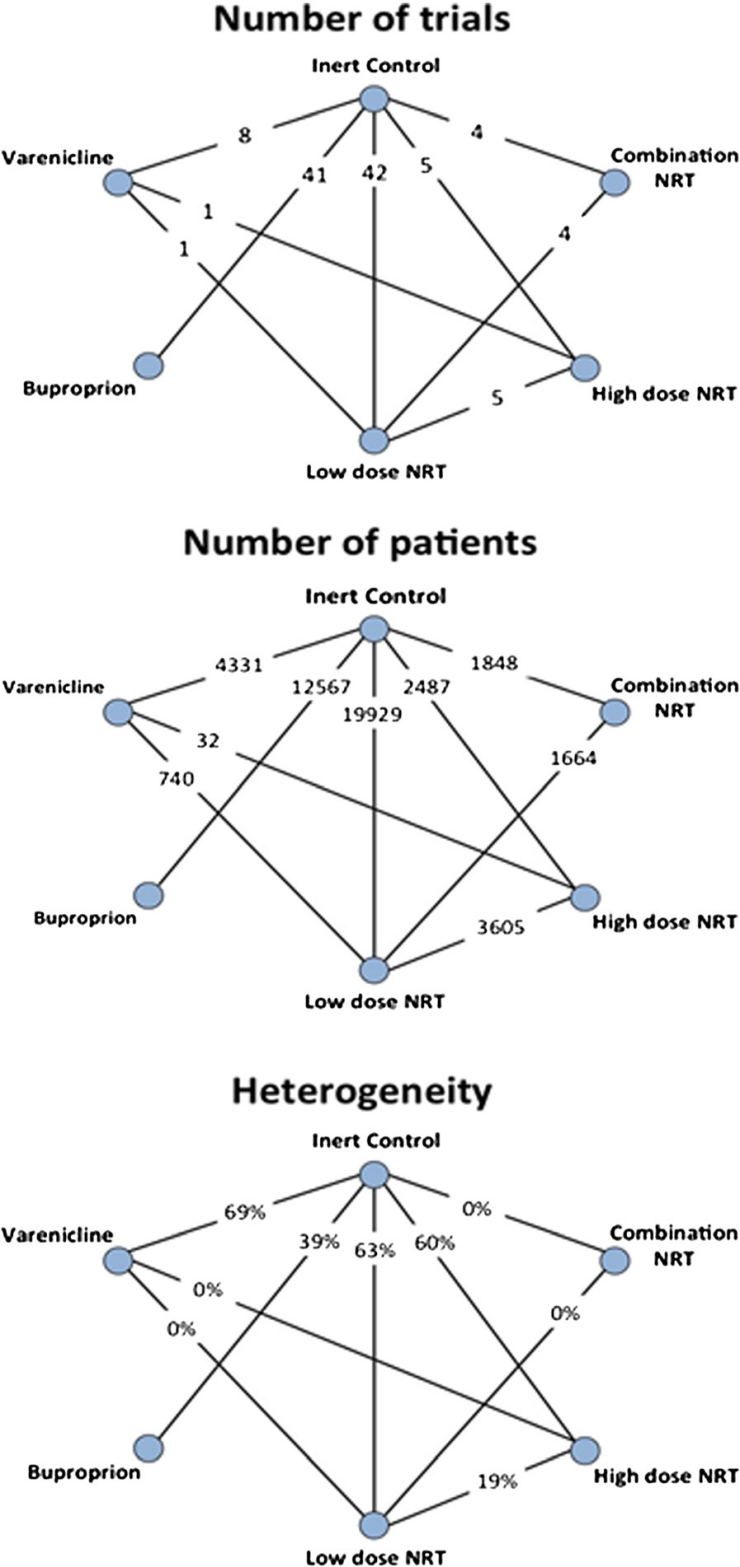
The number of trials, number of patients and degree of heterogeneity (I2) for each comparison in the treatment network that is informed by head-to-head evidence.

**Figure 2:**
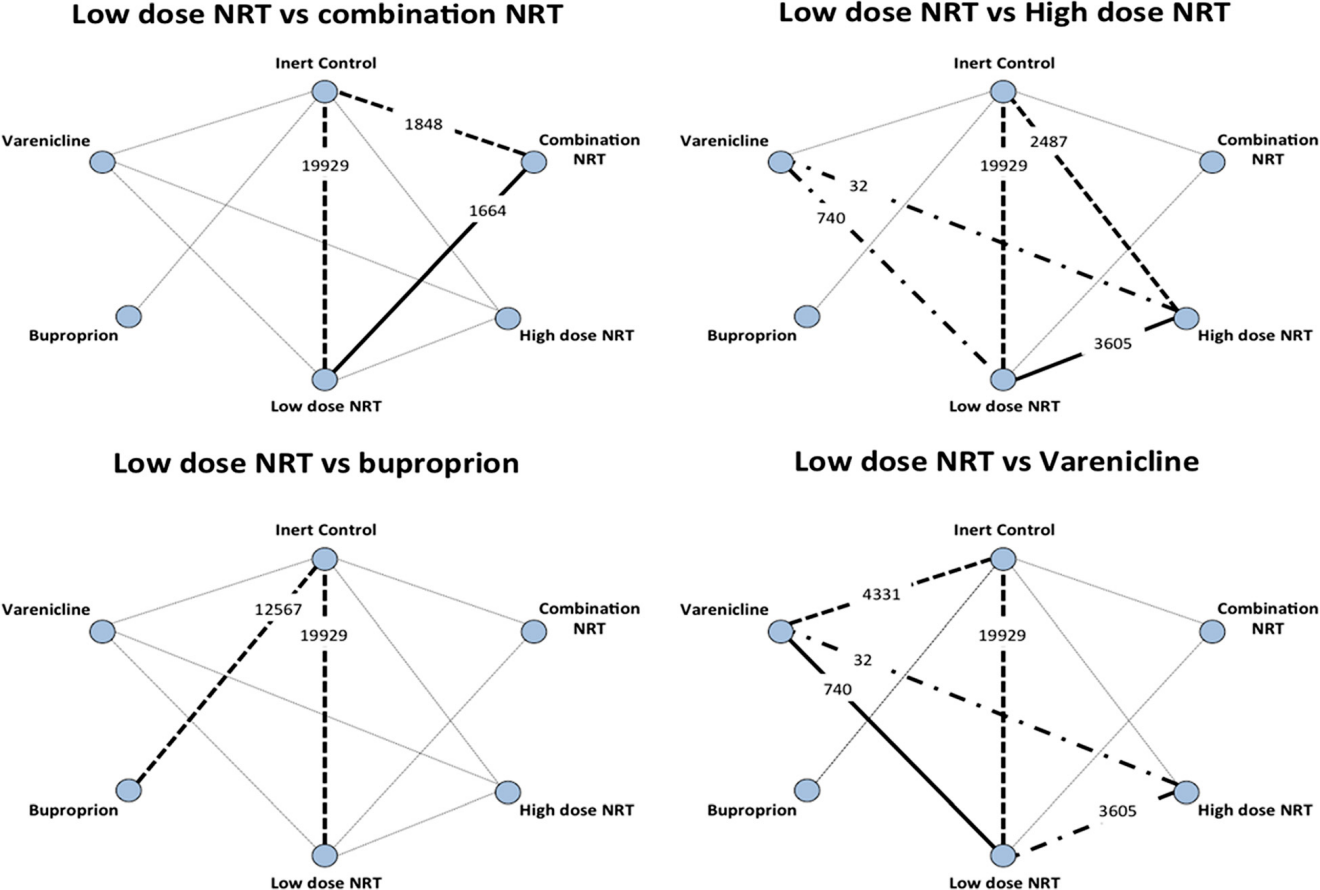
**The sources and strength of direct and indirect evidence (by crude sample size) for the four comparisons of newer treatments versus low-dose NRT.** The thickened solid lines indicate the direct evidence for the comparison of interest. The thickened dashed lines indicate the indirect evidence that adds to the total effective sample size. The thickened dot-dashed lines indicate a (sparse) source of indirect evidence that can be ignored.

The median smoking cessation success rate at 6 months for patients in the inert control group is 15.0% across the 79 included trials reporting at this time point. Assuming a 1.5 relative risk, we would expect a 22.5% success rate with low-dose NRT. An additional 20% relative efficacy would suggest a 26.0% success rate (with any of the newer treatments). Accepting a maximum type I error of α = 5% and a power of (1-β) = 90%, the required sample size to demonstrate that any of the newer interventions are at least 20% better than low-dose NRT is 6,303. To assess the strength of the evidence contained in the treatment network, we calculate the effective sample size (number of patients) for each of the newer treatments compared with low-dose NRT. We do this with and without taking heterogeneity into account. We subsequently calculate the information fraction and power based on the effective sample size. Figure 1 presents the number of trials, number of patients and degree of heterogeneity in the comparisons informed by head-to-head evidence. Figure 2 presents the sources of direct and indirect evidence for the four comparisons of interest (newer treatments vs. low-dose NRT).

For low-dose NRT vs. combination NRT, the direct evidence includes 1,664 patients (and no heterogeneity). Indirect evidence exists with inert control as the common comparator. The comparison of low-dose NRT and inert control includes 19,929 patients, but with 63% heterogeneity, so the heterogeneity penalized sample size is 19,929 × (1–0.63) = 7,374. The comparison of combination NRT vs. inert control includes 1,848 patients (and no heterogeneity). The effective indirect sample size without heterogeneity penalization (*n*_*indirect*_) and the effective indirect sample size with heterogeneity penalization (*n*_*indirect-Pen*_) are therefore

$$n_{\mathrm{indirect}}=\left(19929*1848\right)/\left(19929+1848\right)=1691$$

and

$$n_{indirect-Pen}=\left(7374*1848\right)/\left(7374+1848\right)=1478$$

Adding this to the direct evidence sample size, we get an effective total sample size of

$$n_{\mathrm{Total}}=1691+1664=3355$$

and

$$n_{Total-Pen}=1478+1664=3142$$

These two total effective sample sizes correspond to an information fraction of 53% and 50% (half of the require sample size has been accumulated) and a statistical power of 66% and 63%. Table 4 presents the direct effective indirect and total sample sizes and the corresponding information fractions and statistical power for all four comparisons of the newer treatments vs. low-dose NRT. The calculations for the remaining three comparisons are presented in Appendix 2.

**Table 4:** The effective sample sizes and corresponding information fractions and power estimates from the four comparisons of newer treatments vs. low-dose NRT

**Comparison (vs. low-dose NRT)**	**Effective head-to-head sample size**	**Effective indirect sample size**	**Total effective sample size**	**Information fraction**	**Statistical power**	Statistical power to detect observed improvement*	**Network meta-analysis odds ratio estimate (95% credible interval)**
Combination NRT	1,664	1,691	3,355	53%	66%	-	1.05 (0.76 – 1.41)
High-dose NRT	3,605	2,211	5,816	92%	88%	>99%	1.32 (1.11 – 1.57)
Buproprion	-	7,707	7,707	>100%	95%	-	0.99 (0.86 – 1.14)
Varenicline	720	3,625	4,268	68%	76%	>99%	1.38 (1.15 – 1.64)

In the network meta-analysis of interventions for smoking cessation, high-dose NRT and varenicline were both significantly better than low-dose NRT and demonstrated effect estimates larger than the a priori considered minimally important difference. For these comparisons, high-dose NRT is supported by 88% power, and varenicline is supported by 76% power. Considering that the true effects of high dose NRT and varenicline over low dose NRT are much higher than the 20% increase that was assumed for these calculations, the true power of these comparisons are also much higher. For example, if the MTC actual effects for varenicline (i.e., a 38% increase in smoking cessation compared with low dose NRT) are used, the statistical power to detect this difference then exceeds 99%. Combination NRT and buproprion both yielded effect estimates very similar to low-dose NRT. Considering that the two are supported by 66% and 95% power, respectively, and that none of the two effect estimates appear superior, it should be reasonable to infer that the two interventions do not offer any noteworthy benefit over low-dose NRT.

## Conclusions

In this article we have outlined available methods for gauging the strength of the evidence in a network meta-analysis using sample size and power considerations. We recommend sample size considerations in the context of the number of patients, as the required calculations are relatively straightforward and will resonate well with most clinicians and decision-makers. The methods we have outlined are of high value to regulatory agencies and decision makers who must assess the strength of the evidence supporting comparative effectiveness estimates.

## Appendix

1.a Calculating the effective number of trials

Consider the situation where three treatments, A, B and C, have been compared head to head in randomized clinical trials. For any one trial, assume that the estimated treatment effect has variance *v.* For a meta-analysis of *2k* trials, using the inverse variance approach would produce an estimated variance of the pooled treatment effect of *σ*^*2*^*/2k.* By the expected variance of an indirect comparison, if we have two comparison including *k* trials, we would expect an indirect variance estimate of *σ*^*2*^*/k + σ*^*2*^*/k = 2σ*^*2*^*/k.* Now letting *R* denote a ratio describing the relationship between the precision of indirect and direct evidence; we can derive *R* as follows

$$R*\left(v/2k\right)=2v/k$$

R=4

That is, in the scenario where the number of trials are equal in the two comparisons informing the indirect comparison (and the other above assumptions are met), it would require four trials in the indirect evidence to produce the same precision as that corresponding to a single head-to-head trial. We can generalize this ratio to the situation where the number of trials is not equal in the two comparisons informing the indirect evidence. Let *k*_*AC*_ and *k*_*BC*_ be the number of trials informing the comparison of A vs. C and B vs. C, respectively. For a single meta-analysis, with *k*_*AC*_ *+ k*_*BC*_ trials we would expect a variance of the pooled effect of *σ*^2^*/(k*_*AC*_ *+ k*_*BC*_*).* Moreover, we would expect a variance from the indirect comparison of *σ*^2^*/k*_*AC*_ *+ v/k*_*BC*_*.* Proceeding as above we then have

$$R.\;\left(v/\left(k_{\mathrm{AC}}+k_{\mathrm{BC}}\right)\right)=v/k_{\mathrm{AC}}+v/k_{\mathrm{BC}}$$

$$R.\;\left(v/\left(k_{\mathrm{AC}}+k_{\mathrm{BC}}\right)\right)=v.\;\left(k_{\mathrm{BC}}+k_{\mathrm{AC}}\right)/\left(k_{\mathrm{BC}}.k_{\mathrm{AC}}\right)$$

$$R={\left(k_{\mathrm{BC}}+k_{\mathrm{AC}}\right)}^{2}/\left(k_{\mathrm{BC}}.k_{\mathrm{AC}}\right)$$

This formula creates the basis for the results presented in Table 1.

1.b Calculating the effective number of patients

Consider the situation where three treatments, A, B and C, have been compared head to head in randomized clinical trials. Assume that the population variance of comparative treatment effects is the same for A vs. B, A vs. C and B vs. C, and assume the population variance produced by a fixed-effect pairwise meta-analysis can be regarded as a large well-designed clinical trial. Let *n*_*AB*_, *n*_*AC*_ and *n*_*BC*_ denote the meta-analysis sample size (total number of patients) for the three comparisons A vs. B, A vs. C and B vs. C, respectively.

We are interested in finding the ratio between the variance of the direct meta-analysis pooled treatment effect estimate and the variance of the indirect meta-analysis pooled treatment estimate. Let *R* denote this ratio, and let *σ*_*AB*_^*2*^, *σ*_*AC*_^*2*^ and *σ*_*BC*_^*2*^ denote the population variances for the three comparisons (where we assume *σ*_*AB*_^*2*^ = *σ*_*AC*_^*2*^ = *σ*_*BC*_^*2*^ = *σ*^*2*^). Then we have

$$\sigma^{2}/\left(R.\left(n_{\mathrm{AC}}+n_{\mathrm{BC}}\right)\right)=\sigma^{2}/n_{\mathrm{AC}}+\sigma^{2}/n_{\mathrm{BC}}$$

$$1/\left(R.\left(n_{\mathrm{AC}}+n_{\mathrm{BC}}\right)\right)=\left(n_{\mathrm{AC}}+n_{\mathrm{BC}}\right)/\left(n_{\mathrm{AC}}.n_{\mathrm{BC}}\right)$$

$$R=\left(n_{\mathrm{AC}}.n_{\mathrm{BC}}\right)/({\left(n_{\mathrm{AC}}+n_{\mathrm{BC}}\right)}^{2}$$

Thus, by multiplying this ratio with the total indirect sample size (n_AC_ + n_BC_) we have that the formula for the effective indirect sample size is

$$n=\left(n_{\mathrm{AC}}.n_{\mathrm{BC}}\right)/\left(n_{\mathrm{AC}}+n_{\mathrm{BC}}\right)$$

When heterogeneity exists for one or both of the comparisons in the indirect evidence, one can penalize the sample size by multiplying by the ‘lack of homogeneity,’ much similar to what is done for a heterogeneity correction of a required meta-analysis sample size. With estimates of the percentage of variation in the meta-analysis due to between-trial heterogeneity for A vs. C, *I*_*AC*_^*2*^*,* and for B vs. C, *I*_*BC*_^*2*^*,* we can derive penalized sample sizes within each comparison

$$n_{AC-Pen}=n_{\mathrm{AC}}.\left(1-{I_{\mathrm{AC}}}^{2}\right)$$

$$n_{BC-Pen}=n_{\mathrm{BC}}\left(1-{I_{\mathrm{BC}}}^{2}\right)$$

and subsequently use these penalized sample sizes in the formula for the effective indirect sample size.

2. Information fraction and power calculations – worked example

For low-dose NRT vs. high-dose NRT, the direct evidence includes 3,605 patients (and no heterogeneity). Indirect evidence exists with inert control as the common comparator. The comparison of low-dose NRT and inert control includes 19,929 patients, but with 63% heterogeneity, so the heterogeneity penalized sample size is 19,929×(1–0.63) = 7,373. The comparison of high-dose NRT vs. inert control includes 2,487 patients, but with 60% heterogeneity, so the heterogeneity penalized sample size is 2,487×(1–0.60) = 1,492. The effective sample size from this indirect comparison is therefore

$$n_{\mathrm{indirect}}=\left(19929\times 2487\right)/\left(19929+2487\right)=2211$$

and

$$n_{indirect-Pen}=\left(7373\times 995\right)/\left(7373+995\right)=877$$

A second indirect comparison with varenicline as the common comparator only includes 32 patients in one of the two involved comparisons. The effective sample size of this indirect comparison (*n*_*indirect*_ = 31) is so comparably small that we choose to ignore it. Adding the above calculated indirect sample sizes to the direct evidence sample size, we get effective total sample sizes of

$$n_{\mathrm{Total}}=3605+2211=5816$$

and

$$n_{Total-Pen}=3605+877=3797$$

This total effective sample sizes correspond to information fractions of 92% and 60% and statistical power estimates of 88% and 72%.

For low-dose NRT vs. buproprion, no direct evidence exists. Indirect evidence exists through inert control as the common comparator. As above, the sample size for low-dose NRT vs. inert control is 19,929, or 7,373 if heterogeneity penalized. The sample size for buproprion vs. inert control is 12,567, or 12,567×(1–0.39) = 7,666 when heterogeneity is penalized. Therefore, the total effective sample sizes (which are equal to the effective indirect sample sizes) are

$$n_{\mathrm{Total}}=n_{\mathrm{indirect}}=\left(19929\times 12567\right)/\left(19929+12567\right)=7707$$

and

$$n_{Total-Pen}=n_{indirect-Pen}=\left(7373\times 7666\right)/\left(7373+7666\right)=3758$$

This total effective sample sizes correspond to information fractions of >100% and 60% and statistical power estimates of 95% and 71%.

For low-dose NRT vs. varenicline, the direct evidence includes 740 patients (and no heterogeneity). As above, the sample size for low-dose NRT vs. inert control is 19,929, or 7,373 if heterogeneity is penalized. The sample size for varenicline vs. inert control is 4,331, or 4,331 × (1–0.69) = 1,343 if heterogeneity is penalized. Therefore, the total indirect sample sizes are

$$n_{\mathrm{indirect}}=\left(19929\times 4331\right)/\left(19929+4331\right)=3558$$

and

$$n_{indirect-Pen}=\left(7373\times 1343\right)/\left(7373+1343\right)=1136$$

and so the total effective sample are

$$n_{\mathrm{Total}}=740+3558=4268$$

and

$$n_{Total-Pen}=740+1136=1876$$

All power, and information fraction calculations above are geared to detect an assumed relative improvement in smoking cessation of 20%. All calculations are highly sensitive to the assumed relative improvement. In particular, assuming larger improvements would result in substantially larger power and information fraction estimates.

## Competing interests

The authors declare that they have no competing interests.

## Authors’ contributions

KT conceived the idea, mathematically derived the proposed formulas, drafted the first manuscript and performed all statistical analyses. EM contributed to the methodological development, writing of the manuscript and interpretation of results. Both authors read and approved the final manuscript.
